# Robotic-assisted technique in plastic and reconstructive surgery: Experience in Taiwan

**DOI:** 10.1016/j.jham.2025.100332

**Published:** 2025-07-25

**Authors:** Yi-Chia Chen, Ming-Hsien Chung, Chieh-Kai Chang, Cheng-Yeu Wu, Yi-Ling Lin, Yueh-Chi Tsai, Chen-Te Lu, I-Chen Chen, Chih-Sheng Lai

**Affiliations:** a Division of Plastic and Reconstructive Surgery, Department of Surgery, Taichung Veterans General Hospital, No. 1650, Sec. 4, Taiwan Blvd., Xitun District, Taichung City, 40705, Taiwan, Republic of China; b Department of Nursing, Hungkuang University, No. 1018, Huanchung Rd., Shalu District, Taichung City, 433304, Taiwan, Republic of China; c Department of Post-Baccalaureate Medicine, National Chung Hsing University, No. 145, Xingda Rd., South District, Taichung City, 402202, Taiwan, Republic of China; d Faculty of Medicine, National Yang Ming Chiao Tung University, School of Medicine, No. 155, Sec. 2, Linong St., Beitou District, Taipei City, 112304, Taiwan, Republic of China; e Division of Plastic Surgery, Chung Shan Medical University Hospital, No. 110, Sec. 1, Jianguo N. Rd., South District, Taichung City, 40201, Taiwan, Republic of China

**Keywords:** Robotics, Robotic-assisted platforms, Da Vinci surgical system, Microsurgery, Reconstructive surgery, Review

## Abstract

**Background:**

Robotic-assisted surgery (RAS) has increasingly been applied in the field of plastic and reconstructive surgery, offering enhanced precision, reduced invasiveness, and improved patient outcomes. This review summarizes Taiwan's pioneering experience with robotic-assisted techniques in this specialty, with an emphasis on clinical applications, educational models, and future directions.

**Methods:**

A narrative literature review was conducted focusing on robotic-assisted plastic and reconstructive surgery with relevance to Taiwanese clinical practice. Articles published between January 2000 and April 2024 were considered. Searches were performed in PubMed, Google Scholar, and Cochrane Library using the keywords: “robotic surgery,” “plastic surgery,” “reconstructive surgery,” “Taiwan,” and “microsurgery.” Inclusion criteria were: (1) studies involving robotic-assisted surgical techniques, (2) relevance to plastic and reconstructive surgery, (3) Taiwanese institutional or clinical context, and (4) availability of clinical or technical outcome data. Articles not published in English, lacking original data, or unrelated to the Taiwan context were excluded.

**Results:**

Robotic-assisted techniques were successfully applied in various reconstructive domains, notably in microsurgical anastomosis for free flap procedures, nerve transfers, and minimally invasive mastectomies. These approaches demonstrated favorable outcomes in terms of operative precision, reduced complications, and patient satisfaction. Taiwan's major medical centers, including Chang Gung Memorial Hospital and Taichung Veterans General Hospital, have been instrumental in driving these innovations. Furthermore, robotic training models facilitated the learning curve for microsurgeons and supported the integration of robotic platforms into surgical education.

**Conclusions:**

Taiwan's experience underscores the feasibility and clinical value of robotic-assisted techniques in plastic and reconstructive surgery. Robotic platforms not only improve surgical outcomes but also expand the scope of reconstructive options. Ongoing research and educational efforts are crucial to optimizing technique standardization and surgeon training in this rapidly evolving field.

## 1 Introduction

Robotic-assisted surgery represents a groundbreaking advancement in the field of surgical medicine, offering unprecedented precision, control, and outcomes in complex procedures. This innovative approach leverages robotic technology to enhance the capabilities of surgeons, enabling them to perform intricate surgeries with greater accuracy and reduced risk of complications.^1^, ^2^, ^3^, ^4^, ^5^, ^6^, ^7^, ^8^, ^9^, ^10^, ^11^ The integration of robotics into plastic and reconstructive surgery is particularly significant due to the meticulous nature of these procedures, which often require the manipulation of delicate tissues and structures. Taiwan has been an active contributor to this growing field. Leading medical centers such as Chang Gung Memorial Hospital and Taichung Veterans General Hospital have implemented robotic-assisted approaches across various subspecialties, including breast reconstruction, head and neck free flap transfers, and peripheral nerve surgery. These national experiences not only demonstrate the feasibility of robotic techniques in microsurgery but also offer important insights into their integration in clinical and educational settings.

The advent of robotic-assisted surgery has introduced several key benefits to plastic and reconstructive practices.^12^, ^13^, ^14^, ^15^, ^16^, ^17^ One of the most notable advantages is the minimally invasive nature of these procedures.^13^^,^^14^ Robotic systems enable surgeons to perform operations through smaller incisions, which translates to reduced trauma to the patient's body, decreased postoperative pain, shorter hospital stays, and faster recovery times.^15^^,^^16^ This minimally invasive approach is particularly advantageous in reconstructive surgeries where preserving the integrity of surrounding tissues is crucial for aesthetic and functional outcomes.

Furthermore, robotic-assisted surgery has shown promising results in terms of improving surgical outcomes and patient satisfaction. Studies have indicated that the use of robotic systems can lead to more consistent and reproducible results, reducing the variability associated with human performance. This consistency is vital in reconstructive surgery, where precise tissue manipulation and alignment are critical for successful outcomes. For instance, in breast reconstruction following mastectomy, robotic-assisted techniques have been demonstrated to enhance the precision of tissue placement and symmetry, contributing to better cosmetic results and patient confidence.^18^, ^19^, ^20^, ^21^

The aim of this review is to explore the role of robotic surgical systems in plastic and reconstructive surgery in Taiwan and assess the outcomes. This review will outline the current applications of robotic-assisted surgery in this field and identify the challenges that need to be addressed for future advancements. This article presents a **narrative review** of robotic-assisted techniques in plastic and reconstructive surgery, with a focus on Taiwan's clinical and educational experiences.

## 2 Animal or cadaver studies on robotic-assisted plastic and reconstructive surgery training

Animal and cadaver studies have been pivotal in advancing the field of robotic-assisted plastic and reconstructive surgery.^22^, ^23^, ^24^, ^25^, ^26^, ^27^, ^28^, ^29^, ^30^ These studies not only provide a platform for evaluating the feasibility and safety of new techniques but also serve as an essential training ground for surgeons. The Da Vinci Surgical System, a widely used robotic platform, has been instrumental in these training studies, allowing for the development of complex surgical skills in a controlled environment before human application.

One prominent study involved the use of a porcine model to train surgeons in the robotic harvesting of the rectus abdominis muscle.^31^ This study demonstrated that the Da Vinci robot could be effectively used to perform this procedure with minimal invasiveness, reducing the associated morbidity seen in traditional open surgical approaches. The porcine model allowed for the simulation of the entire surgical process, including the installation of the robot, surgical approaches with trocars, and the dissection and collection of the muscle. Results indicated a clear learning curve, with improved efficiency and outcomes over successive trials.

In another study, a cadaver model was utilized to explore the feasibility of endoscopic, robot-assisted C7 nerve root transfer from the contralateral healthy side.^32^ This minimally invasive technique involved the use of the Da Vinci robot to transfer the C7 nerve root without the need for a graft, thereby reducing the potential for donor site morbidity and extensive scarring. The study verified the hypothesis that a direct retropharyngeal suture could be achieved using this robotic system, demonstrating the potential for improved surgical outcomes in complex nerve reconstruction procedures.

These animal^22^, ^23^, ^24^, ^25^, ^26^, ^27^ and cadaver^28^, ^29^, ^30^ studies underscore the significant advantages of robotic-assisted systems in plastic and reconstructive surgery. They provide a safe and effective means for surgeons to hone their skills and refine techniques, thereby enhancing the precision and success rates of actual surgical procedures. Moreover, the use of these models addresses ethical considerations by adhering to the principle of “never the first time on a patient,” ensuring that new surgical methods are thoroughly vetted and practiced before human application.

## 3 Head and neck reconstructive surgery

Robotic-assisted surgery has advanced head and neck reconstructive surgery, offering enhanced precision, reduced invasiveness, and improved patient outcomes. The Da Vinci Surgical System, a leading robotic platform, has been instrumental in these advancements. This system provides high-definition, three-dimensional visualization and articulated instruments that enable precise and stable movements, minimizing the technical difficulties often encountered in conventional surgical procedures.

### 3.1 International perspective

Robotic-assisted head and neck reconstructive surgery has shown promising results, particularly in the context of oropharyngeal reconstruction. Studies have demonstrated that the use of the Da Vinci Surgical System allows for complex surgical procedures without the need for extensive incisions such as mandibulotomy or lip-split incisions, which are typically required in traditional methods. For instance, Selber et al.^13^ reported successful oropharyngeal reconstructions using the Da Vinci system, highlighting the benefits of avoiding mandible splitting, which significantly reduces postoperative morbidity and improves cosmetic outcomes.

A study by Mukhija et al.^31^ also supports these findings, demonstrating that transoral robotic-assisted free flap reconstruction can be performed with high success rates and minimal complications. The robotic system allows surgeons to operate in confined spaces with greater dexterity and control, enhancing the accuracy of tissue manipulation and anastomosis. Furthermore, international clinical trials have consistently shown that robotic-assisted surgeries result in shorter hospital stays, faster recovery times, and better functional outcomes compared to traditional open surgeries.

### 3.2 Domestic perspective (Taiwan)

In Taiwan, the application of robotic-assisted surgery in head and neck reconstruction has been expanding significantly. Studies conducted at major medical centers, such as Taichung Veterans General Hospital, have demonstrated the efficacy of the Da Vinci Surgical System in performing free flap reconstructions for oropharyngeal cancer patients. Lai et al.^32^^,^^33^ reported a series of cases in which the Da Vinci system was employed for transoral robotic-assisted free flap reconstructions. The results showed that the robotic system provided a clear, magnified view and enabled precise instrument movements, resulting in successful surgical outcomes without flap failures or significant complications. Additionally, the system has proven capable of performing microvascular anastomosis for vessels larger than 1 mm, suggesting its potential to replace traditional microscopes in future microsurgical procedures.

In another study, Tsai et al.^34^ compared the functional outcomes and complications of robot-assisted versus conventional free flap reconstructions. The findings revealed that patients undergoing robotic-assisted procedures had superior postoperative functional outcomes, including better oral intake and speech capabilities, compared to those who underwent conventional surgeries. The robotic system's ability to perform minimally invasive procedures without the need for extensive incisions contributed to these improved outcomes.

Overall, both international and domestic experiences highlight the transformative impact of robotic-assisted surgery in head and neck reconstruction. The Da Vinci Surgical System has enabled surgeons to perform complex procedures with enhanced precision and control, resulting in better patient outcomes and reduced postoperative complications.

## 4 Nerve reconstructive surgery

Nerve reconstructive surgery has significantly advanced with the advent of robotic-assisted techniques, particularly using the Da Vinci Surgical System. These advancements have enabled more precise and minimally invasive procedures, which are crucial for the delicate nature of nerve surgeries.

### 4.1 International perspective

Internationally, robotic-assisted nerve reconstructive surgery has shown great promise. A study by Liverneaux et al.^35^ demonstrated the feasibility of using the Da Vinci robot for the Oberlin procedure, which involves nerve transfer to restore elbow flexion in patients with brachial plexus injuries. In particular, the use of the Da Vinci system has enhanced the precision and outcomes of microsurgical suturing of nerves. The study reported successful outcomes with minimal complications, showcasing the potential of robotic systems to improve the precision and efficacy of nerve surgeries.

### 4.2 Domestic perspective (Taiwan)

In Taiwan, the use of robotic-assisted techniques in nerve reconstructive surgery is also gaining traction. A notable study led by Chang et al.^36^, ^37^, ^38^ involved the use of the Da Vinci robot for sympathetic nerve reconstruction using autologous sural nerve grafts. The results showed that the Da Vinci system allowed for precise suturing in confined anatomical spaces, which is critical for successful nerve regeneration. This technique has been particularly beneficial in addressing complications such as compensatory sweating following endoscopic thoracic sympathectomy The study demonstrated the technical feasibility and clinical success of this approach, with all procedures being successfully completed without conversion to open surgery. The robotic system's precise control and three-dimensional visualization were crucial in achieving these outcomes.

Additionally, researchers form Taiwan have been exploring the use of robotic systems for peripheral nerve surgeries. A systematic review by Chen et al.^39^ highlighted the growing applications of robotic-assisted techniques in peripheral nerve reconstruction, including brachial plexus reconstruction and nerve decompression. The review emphasized that while robotic-assisted surgery is still in its early stages in this field, the potential benefits, including reduced invasiveness and improved precision, are significant.

Overall, both international and domestic experiences indicate that robotic-assisted nerve reconstructive surgery, facilitated by systems like the Da Vinci Surgical System, offers enhanced precision, reduced invasiveness, and improved clinical outcomes. As research and clinical experience grow, these techniques are expected to become more widely adopted, further advancing the field of nerve surgery.

## 5 Implant-based breast reconstruction

Implant-based breast reconstruction has become a widely practiced approach in breast surgery, significantly enhanced by the introduction of robotic-assisted techniques such as the Da Vinci Surgical System. This system allows for precise, minimally invasive procedures, leading to improved cosmetic outcomes and reduced complications.

### 5.1 International perspective

Globally, the adoption of robotic-assisted implant-based breast reconstruction has been growing.^40^, ^41^, ^42^ The Da Vinci Surgical System has been instrumental in performing nipple-sparing mastectomies (NSM) followed by immediate implant reconstruction. Studies have demonstrated that robotic-assisted NSM (R-NSM) offers superior cosmetic results, reduced scarring, and improved preservation of the nipple-areola complex compared to conventional methods. For example, Sarfati et al.^43^ reported that the Da Vinci Xi system significantly improved the technical aspects of NSM by providing better control and flexibility during surgery, leading to enhanced aesthetic outcomes and lower complication rate.

Moreover, international studies have shown that R-NSM can decrease operative time and hospitalization duration, contributing to faster recovery and higher patient satisfaction. A study by Toesca et al.^18^ highlighted that robotic-assisted procedures could maintain symmetry and reduce the risk of complications such as capsular contracture and implant malposition, even in patients with larger breasts or those requiring bilateral procedures.

### 5.2 Domestic perspective (Taiwan)

In Taiwan, the integration of robotic-assisted techniques in implant-based breast reconstruction is also advancing. Researches by Lai et al.^44^, ^45^, ^46^, ^47^, ^48^, ^49^, ^50^ detailed the outcomes of R-NSM followed by immediate implant reconstruction, demonstrating that the robotic approach offers superior cosmetic results and reduced complication rates compared to traditional and endoscopic-assisted methods.

Another study by Kuo et al.^51^ evaluated the feasibility of robotic-assisted mastectomy followed by immediate autologous microsurgical free flap reconstruction. Although this study focused on autologous reconstruction, the findings underscored the potential of robotic systems to enhance surgical precision and outcomes, suggesting that similar benefits could be applied to implant-based reconstructions.

Furthermore, domestic studies have highlighted the learning curve associated with robotic-assisted breast surgery. Loh et al.^52^ analyzed the learning curve for R-NSM in Taiwan, noting significant improvements in operative times and reduced complications as surgeons gained experience with the robotic system. This study emphasized the importance of specialized training to achieve optimal outcomes in robotic-assisted breast reconstruction.

Overall, both international and domestic experiences indicate that robotic-assisted implant-based breast reconstruction, facilitated by the Da Vinci Surgical System, is a feasible and effective approach. This technique enhances cosmetic outcomes, reduces the risk of complications, and contributes to improved overall patient satisfaction and quality of life.

## 6 Free autologous tissue in breast reconstruction

Free autologous tissue in breast reconstruction has become a cornerstone of breast cancer surgery due to its natural, long-lasting results and fewer complications compared to implant-based reconstruction. This technique involves using the patient's own tissue, such as the Deep Inferior Epigastric Perforator (DIEP) flap or the Profunda Artery Perforator (PAP) flap, to reconstruct the breast. The Da Vinci Surgical System, a state-of-the-art robotic platform, has further refined these procedures, allowing for greater precision and less invasive techniques.

Internationally, robotic-assisted breast reconstruction has shown promising results. In a study conducted by Manrique et al.,^53^ the feasibility of robotic-assisted DIEP flap harvest was confirmed in a cadaver model, showcasing the system's potential to enhance precision and outcomes in clinical settings. The integration of robotic systems like the Da Vinci has improved the accuracy of flap dissection and minimized donor site morbidity.^54^^,^^55^ Studies have demonstrated that the use of robotic systems in DIEP flap harvesting significantly reduces the size of the incision required on the rectus abdominis fascia, which leads to decreased postoperative pain and quicker recovery times. The robotic approach allows surgeons to perform complex dissections through smaller incisions, enhancing the overall aesthetic outcome and patient satisfaction.

In Taiwan, the adoption of robotic-assisted breast reconstruction is also gaining momentum. Hospitals like Chang Gung Memorial Hospital a world-renewned medical center in Taiwan, have been at the forefront of implementing these advanced techniques. A study led by Huang et al.^56^ at this institution explored the feasibility and aesthetic outcomes of using the Da Vinci system for robotic nipple-sparing mastectomy (R-NSM) followed by immediate microsurgical free flap reconstruction. The results indicated that robotic-assisted procedures not only provide excellent oncological safety but also offer superior cosmetic results compared to traditional methods. The precise dissection capabilities of the Da Vinci system allowed for minimal incisions and reduced visible scarring, significantly improving the postoperative appearance.

One of the major benefits of using the Da Vinci system in both international and Taiwanese settings is its ability to facilitate complex microvascular anastomosis through smaller incisions. This capability is crucial for procedures like the DIEP flap, where the blood vessels supplying the flap must be meticulously dissected and connected. The enhanced visualization and dexterity provided by the robotic system reduce the risk of complications such as flap necrosis and improve the overall success rate of the reconstruction.

Despite these advancements, there are challenges to be addressed. The high cost of robotic systems and the need for specialized training for surgeons are significant barriers. Furthermore, the learning curve associated with mastering robotic-assisted techniques can be steep, requiring dedicated time and resources. However, with ongoing research and increasing clinical experience, these obstacles are gradually being overcome, paving the way for more widespread adoption of robotic-assisted breast reconstruction worldwide.

In conclusion, the integration of the Da Vinci Surgical System in free autologous tissue breast reconstruction represents a significant advancement in the field, offering enhanced precision, reduced invasiveness, and improved aesthetic outcomes.^51^^,^^57^ As both international and Taiwanese experiences suggest, the continued evolution of these techniques holds great promise for the future of breast cancer surgery.

## 7 Future perspectives

In addition to its clinical benefits, robotic-assisted plastic and reconstructive surgery also offers educational advantages.^22^^,^^58^, ^59^, ^60^, ^61^ Surgeons in training can benefit from the use of robotic simulators, which provide a safe and controlled environment for developing their skills. These simulators allow for repetitive practice and the refinement of techniques without the immediate pressure and risks associated with live surgery. As a result, surgical trainees can achieve a higher level of proficiency before performing procedures on actual patients.

Despite the numerous advantages, the adoption of robotic-assisted surgery in the field of plastic and reconstructive surgery is not without challenges. The high cost of robotic systems and the need for specialized training can be barriers to widespread implementation. Additionally, the long-term outcomes and cost-effectiveness of these technologies are still being evaluated. However, as the technology continues to evolve and become more accessible, it is expected that the adoption of robotic-assisted techniques will expand, further transforming the landscape of plastic and reconstructive surgery. This literature review provides an overview of the performance of robotic surgery in plastic and reconstructive surgery. The number of studies is limited, and the reported outcomes often lack sufficient comparison between robot-assisted and conventional manual techniques, as well as a lack of statistical analyses. While this review highlights the potential benefits of using robots in plastic and reconstructive surgery, it does not provide sufficient data to confirm these advantages. Further large-scale clinical studies with comprehensive statistical analyses comparing robot-assisted operations to conventional microsurgery are necessary to validate these potential benefits.

Although robotic-assisted surgery offers significant technical advantages, its widespread clinical adoption remains challenged by both economic and educational barriers. The cost of robotic systems, including acquisition, maintenance, and consumables, is substantially higher than that of conventional surgical setups. Additionally, the extended duration of robotic procedures during the initial learning curve may further increase operational costs. However, the steep learning curve observed in the early stages can be mitigated through dedicated training programs and simulation models, which are being increasingly adopted in Taiwan. It is worth noting that publicly available cost-comparison data specific to robotic-assisted plastic and reconstructive surgery in Taiwan remains limited, and further studies are needed to provide a detailed economic evaluation.

In conclusion, robotic-assisted plastic and reconstructive surgery represents a significant technological advancement. By enhancing surgical precision, reducing invasiveness, and improving patient outcomes, robotic systems are poised to become an integral part of modern surgical practice. Continued research and development, along with efforts to address current limitations, will be essential in fully realizing the potential of this innovative approach.

## Funding

The authors received no financial support for the research, authorship, or publication of this article.

## Declaration of competing interest

The authors declare that there are no potential conflicts of interest regarding the research, authorship, or publication of this article.
